# Recruiting Young People for Digital Mental Health Research: Lessons From an AI-Driven Adaptive Trial

**DOI:** 10.2196/60413

**Published:** 2025-01-14

**Authors:** Wu Yi Zheng, Artur Shvetcov, Aimy Slade, Zoe Jenkins, Leonard Hoon, Alexis Whitton, Rena Logothetis, Smrithi Ravindra, Stefanus Kurniawan, Sunil Gupta, Kit Huckvale, Eileen Stech, Akash Agarwal, Joost Funke Kupper, Stuart Cameron, Jodie Rosenberg, Nicholas Manoglou, Manisha Senadeera, Svetha Venkatesh, Kon Mouzakis, Rajesh Vasa, Helen Christensen, Jill M Newby

**Affiliations:** 1 Black Dog Institute University of New South Wales Sydney Australia; 2 Applied Artificial Intelligence Institute Deakin University Melbourne Australia; 3 Centre for Digital Transformation of Health University of Melbourne Melbourne Australia

**Keywords:** recruitment, Facebook, retention, COVID-19, artificial intelligence

## Abstract

**Background:**

With increasing adoption of remote clinical trials in digital mental health, identifying cost-effective and time-efficient recruitment methodologies is crucial for the success of such trials. Evidence on whether web-based recruitment methods are more effective than traditional methods such as newspapers, media, or flyers is inconsistent. Here we present insights from our experience recruiting tertiary education students for a digital mental health artificial intelligence–driven adaptive trial—Vibe Up.

**Objective:**

We evaluated the effectiveness of recruitment via Facebook and Instagram compared to traditional methods for a treatment trial and compared different recruitment methods’ retention rates. With recruitment coinciding with COVID-19 lockdowns across Australia, we also compared the cost-effectiveness of social media recruitment during and after lockdowns.

**Methods:**

Recruitment was completed for 2 pilot trials and 6 minitrials from June 2021 to May 2022. To recruit participants, paid social media advertising on Facebook and Instagram was used, alongside mailing lists of university networks and student organizations or services, media releases, announcements during classes and events, study posters or flyers on university campuses, and health professional networks. Recruitment data, including engagement metrics collected by Meta (Facebook and Instagram), advertising costs, and Qualtrics data on recruitment methods and survey completion rates, were analyzed using RStudio with R (version 3.6.3; R Foundation for Statistical Computing).

**Results:**

In total, 1314 eligible participants (aged 22.79, SD 4.71 years; 1079, 82.1% female) were recruited to 2 pilot trials and 6 minitrials. The vast majority were recruited via Facebook and Instagram advertising (n=1203; 92%). Pairwise comparisons revealed that the lead institution’s website was more effective in recruiting eligible participants than Facebook (*z*=3.47; *P*=.003) and Instagram (*z*=4.23; *P*<.001). No differences were found between recruitment methods in retaining participants at baseline, at midpoint, and at study completion. Wilcoxon tests found significant differences between lockdown (pilot 1 and pilot 2) and postlockdown (minitrials 1-6) on costs incurred per link click (lockdown: median Aus $0.35 [US $0.22], IQR Aus $0.27-$0.47 [US $0.17-$0.29]; postlockdown: median Aus $1.00 [US $0.62], IQR Aus $0.70-$1.47 [US $0.44-$0.92]; W=9087; *P*<.001) and the amount spent per hour to reach the target sample size (lockdown: median Aus $4.75 [US $2.95], IQR Aus $1.94-6.34 [US $1.22-$3.97]; postlockdown: median Aus $13.29 [US $8.26], IQR Aus $4.70-25.31 [US $2.95-$15.87]; W=16044; *P*<.001).

**Conclusions:**

Social media advertising via Facebook and Instagram was the most successful strategy for recruiting distressed tertiary students into this artificial intelligence–driven adaptive trial, providing evidence for the use of this recruitment method for this type of trial in digital mental health research. No recruitment method stood out in terms of participant retention. Perhaps a reflection of the added distress experienced by young people, social media recruitment during the COVID-19 lockdown period was more cost-effective.

**Trial Registration:**

Australian New Zealand Clinical Trials Registry ACTRN12621001092886; https://tinyurl.com/39f2pdmd; Australian New Zealand Clinical Trials Registry ACTRN12621001223820; https://tinyurl.com/bdhkvucv

## Introduction

### Background

Advances in digital clinical trial infrastructure have made fully remote clinical trials more commonplace, particularly in the field of digital mental health. In such trials, participants are screened, assessed, treated, and followed up entirely via remote digital methods. Pivotal to the scientific validity of these trials is the timely and adequate recruitment of target participants who meet a set of prespecified eligibility criteria. Due to the paucity of research into best practice methods for efficiently recruiting trial samples, in this paper, we present insights from our experiences recruiting young participants for a digital mental health trial that used an artificial intelligence (AI)–driven adaptive trial design. This type of trial design has the potential to minimize time, costs, and the sample size needed to achieve sufficient statistical power [1]. Additionally, this method facilitates the optimization of treatment plans, which can enhance adherence and lower attrition [2]. However, compared to traditional randomized controlled trials (RCTs), AI-adaptive trials face an increased risk of insufficient and untimely enrollment due to the need for frequent, short-spanned recruitment drives (eg, monthly recruitment drives over a year). Hence, identifying recruitment methodologies that are both cost-effective and time-efficient is crucial for the success of such trials.

What recruitment methods are currently used and how do they compare with each other? A review by Lane et al [3] on online recruitment methods for web-based and mHealth studies found that successful recruitment of participants into a trial varied widely depending on the population, budget, intervention, costs, and study design. Of the studies included in their review, less than half (42%) found Facebook advertisements to be an effective method of recruitment and a quarter found Google advertisements to be the most effective method [3]. Only 1 study reported better outcomes using traditional methods of recruitment (eg, print advertisements and flyers) [3]. Overall, there was no consistent evidence on whether web-based recruitment methods were more effective than traditional methods such as newspapers, media, or flyers [3]. Similar mixed findings were echoed by a systematic review of recruitment strategies in mental health clinical trials [4], and a scoping review that examined the role of social media in clinical trials recruitment [5].

Specific to online recruitment methods, there is also contradictory evidence regarding the most cost-effective platform. Morgan et al [6] used a range of internet-based methods to recruit for the Mood Memos depression prevention study, including Facebook, emails, search engine advertisements, and forum posts, and reported that advertising on Google was more cost-effective than Facebook advertising (Aus $12 [US $7.46] per recruited participant vs Aus $19.89 [US $12.37] per recruited participant) in signing up participants. Similarly, recruitment costs for a large RCT of web-based smoking interventions were highest for Facebook (US $40.51) per randomized participant, compared to Google (US $34.71), traditional sources such as press releases, newspaper, radio, and television interviews (US $20.30), and using an online survey panel company (US $13.95) [7]. In contrast, Facebook advertising was found to be more cost-effective for recruiting a large sample of general community participants for suicide prevention research (Aus $2.81 [US $1.75] per completed survey) [8]. Similarly, Batterham [9] reported that advertising on a Facebook page that featured links to a survey on suicidal ideation was more effective in yielding completions (Aus $1.51 [US $0.94] per completion), compared to advertisements linked to an external website (Aus $9.82 [US $6.11] per completion). Both were more cost-effective than postal surveys, which averaged Aus $19.10 [US $11.88] per completion [9]. In a review of 176 studies that used social media for mental health research recruitment, Facebook was found to be the most preferred platform, at a median cost of US $19.47 per final recruited study participant [10]. The authors also found that in 2 out of 3 studies included in the review, social media recruitment performed as well as or better than traditional methods in the number and cost of final enrolled participants [10].

Differences in effectiveness between recruitment methods can often be difficult to interpret. In their systematic review, Liu et al [4] found that it was difficult to assess the overall effectiveness of any particular recruitment strategy as some strategies that worked well for a particular population may not work as well for others. For example, the greater costs of Facebook advertising for the Mood Memos study relative to the other studies described may be due to study requirements to target adults with subthreshold depression symptoms as opposed to recruiting a nonclinical sample. In terms of recruitment of young participants, evidence suggests that online advertisements are effective at appealing to this particular demographic. For example, Ford et al [11] examined paid advertising on Instagram, Snapchat, and Facebook to recruit youths (aged 13-20 years) for 2 cross-sectional surveys. They concluded that these platforms appear to be a modern and cost-effective approach to reaching youths with surveys on sensitive health topics [11]. Similar findings were obtained from an Australian health survey study that assessed the feasibility of recruiting young females using targeted advertising on Facebook [12]. In another study, Lee et al [8] found that male participants were more difficult to recruit compared to female participants but were able to use targeted, gender-specific advertisements (different language and imagery compared to gender-neutral advertisements) on Facebook to recruit more males. This ability to target different subgroups and easily make changes to targeting strategies are significant benefits of Facebook recruitment. The impact of different targeting strategies can be monitored (eg, costs per click), and further tweaks can be made to determine the best way to reach the target population. In addition, marketing collaterals such as images, headlines, and copy can be changed in order to find the most effective study advertisement [7].

Overall, these studies suggest that online advertising via platforms such as Facebook and Instagram is promising for the effective recruitment of young adults for mHealth survey studies. However, evidence supporting using these recruitment strategies for treatment studies is scarcer. A recent Danish study demonstrated the feasibility of using Facebook and Instagram to recruit young Danes (aged 15-25 years) for an RCT to evaluate a national online youth mental health promotion service [13]. A study that assessed the effectiveness of social media (Facebook), targeted mailing, and in-person recruitment of young adults for diabetes self-management clinical trials found that leveraging a variety of recruitment strategies produced a more representative sample of young adults [14]. To our knowledge, no single study has investigated the effectiveness of various recruitment methods to enlist distressed young adults in digital intervention studies.

### The Vibe Up Trial

Vibe Up is an AI-driven adaptive trial, in which a series of “mini” trials are performed instead of 1 large trial. AI methods are used to update an underlying model of the interventions’ effectiveness and alter the proportion of participants allocated to each intervention in the next minitrial. Under this scheme, progressively fewer participants are allocated to less effective interventions in later minitrials, with the aim of identifying the most effective intervention as quickly as possible. This trial delivered brief interventions, including mindfulness (a set of five 3-5 minute instructor-guided audio meditations), physical activity (an evidence-based 7-minute high-intensity circuit training protocol, or self-selected daily physical activity), sleep hygiene (a program of 4 infographic-based information modules), and ecological momentary assessment (blended protocol consisting of signal contingent and event-contingent), to ease psychological distress in Australian tertiary education students [15]. A mobile app, Vibe Up, was purpose-built for these trials, allowing eligible students to participate in a “minitrial” by downloading the app from the App Store or Google Play. To ensure that data was captured as intended and to detect fraudulent activities, data verification was carried out by the data manager after the completion of each minitrial. Recruitment for Vibe Up coincided with COVID-19 lockdowns in Australia. This provided us with the opportunity to assess the cost-effectiveness of social media recruitment during a pandemic compared to its aftermath. Research has shown significant increases in distress among young people as a result of the COVID-19 pandemic [16-18]. Transition to online learning for students and restrictions on routine daily activities affected the well-being of young people [19-21]. With access to in-person mental health support no longer possible, we examined whether the cost-effectiveness of recruitment differed between the lockdown and the postlockdown period.

### Aims

This study evaluated recruitment data of an AI-driven adaptive digital mental health trial aimed at reducing psychological distress in Australian tertiary education students [15]. This study aims to (1) evaluate the effectiveness of recruitment via Facebook and Instagram compared to traditional methods for a treatment study, (2) compare the cost-effectiveness of online recruitment during and after COVID-19 lockdown, and (3) compare retention rates of different recruitment methods used in this study.

## Methods

### Study Design

This study evaluated recruitment data collected from the Vibe Up study [15]. Data included engagement metrics collected by Meta (Facebook and Instagram), advertising costs, and Qualtrics data [22] on recruitment methods and rates of survey completion. To recruit participants, paid social media advertising on Facebook and Instagram was used, alongside mailing lists of university networks and student organizations or services, media releases, announcements during classes and events, study posters or flyers on university campuses, and our health professionals’ network. Recruitment for 2 pilot trials and 6 minitrials occurred between June 2021 and May 2022.

### Participants

Participants were Australian tertiary education students. No restrictions were placed on the state or territory of origin. In order to participate in the study, participants must (1) be aged 18 years or older; (2) be currently enrolled as a student at an Australian university, technical and further education, or other higher education institution; (3) be currently residing in Australia and planning to be a resident throughout the study period; (4) be an advanced, fluent, or native English speaker; (5) own an eligible personal smartphone (iPhone 6S or Android 5 or later) with active mobile number and internet access; and (6) score 20 or more on the Kessler Psychological Distress Scale, 10-item version [23].

Participants are excluded if they (1) score 21 or more on the Suicide Ideation Attributes Scale [24]; (2) report a current active diagnosis of psychosis or bipolar disorder; (3) have already participated in a “minitrial” in the study; (4) indicate major disruptions or events in the next 2 months which may make it difficult to take part in the study; (5) indicate plans to travel outside of Australia in the next 2 months; and (6) indicate that they would be unable to safely undertake a physical activity intervention if allocated to receive this treatment.

### Procedure

Recruitment was conducted by the research team in collaboration with the lead institution’s in-house marketing and communications department. A social media targeting strategy was developed (Table 1). Clicking on the Meta advertisements (Facebook and Instagram) took potential participants to a landing page, hosted on the lead research institution’s website, which contained study information and a direct link to the screening survey that was administered on the Qualtrics XM survey platform [22]. The recruitment window for each Vibe Up trial remained open for up to 7 days only; however, to not risk losing any potential participants between recruitment drives, the survey link was replaced by an expression of interest form on the website to capture the name, email address, and education institution of interested students. These students were contacted at the beginning of the following recruitment drive. Two pilot trials were carried out before recruitment began for 6 minitrials.

**Table 1:** Profile of individuals targeted on social media.

Category	Detail
Location	Australia
Age (years)	18-40
Sex	Male or female
Education	At university (undergraduate) or at university (postgraduate)
Interests	Science, artificial intelligence, mindfulness, computer science, *DSM-5*^a^, mental health foundation, Headspace, Beyond Blue or Lifeline (crisis support service), and international students

^a^DSM-5: Diagnostic and Statistical Manual of Mental Disorders (Fifth Edition).

Active recruitment was conducted via paid advertising on Facebook and Instagram. Traditional methods for recruiting students such as mailing lists, study flyers or posters, class or event announcements, and posts on the Black Dog Institute website and university media were also used. Meta’s “dynamic creative” function was used to upload a variety of images, copies, and headlines. Meta delivered a combination of these elements until it determined the top-performing combination and the combination that delivered on the objective at the cheapest cost.

We trialed different advertising objectives, using Meta’s (Facebook and Instagram) preset choices. For example, the use of link clicks allowed Meta to deliver study advertisements to people most likely to click on them, with the aim of obtaining the cheapest cost per click. We then unsuccessfully trialed a “landing page view objective” which focused on getting people to not just click on the advertisement, but to load and engage with the content on the page. To improve performance, for minitrials 5 and 6, the Meta pixel was used to set up an “initiate checkout” conversion on the “GET STARTED” button which appeared at the bottom of the recruitment website and took participants to the Qualtrics screening survey. In this instance, Meta optimized study advertisements toward people most likely to take this action.

### Measures

Metrics used to measure recruitment in this study were derived from Facebook analytics data and Qualtrics survey data. For ease of comparison, metrics were largely based on previous research in this area [8,9] and are included in Table 2.

**Table 2:** Study measures and definitions.

Variable name	Definition
Amount spent on advertising per trial (Aus $)	Facebook and Instagram advertising costs per trial in Australian dollars.
Impressions	Number of times study advertisements were viewed.
Reach	Number of people that viewed study advertisements.
Link clicks	Number of times that links to study advertisements were clicked on.
Unique link clicks	Number of people that clicked on study advertisement links, regardless of how many times they clicked on a link.
Click through rate	The percentage of people who viewed and then clicked on the study advertisement. This was calculated by dividing the total number of clicks by the total number of impressions multiplied by 100. Click-through rates measure how successful the study advertisements are in capturing people’s attention.
Number of students screened	Number of people who completed screening.
Number of eligible students	Number of people who were eligible to take part in the study.
Number of students who downloaded the study app	Number of people who downloaded the study app from Google Play or the App Store.
Number of students who completed a trial	Number of people who completed the 3 phases of the study (baseline, midpoint, and post).
Costs per screened	Costs per person who completed the screening questionnaires in Australian dollars.
Costs per eligible	Costs per person who was eligible to participate in the study in Australian dollars.
Costs per downloaded	Costs per person who downloaded the study app from Google Play or the App Store in Australian dollars.
Costs per completed	Costs per person who completed all three phases of the study in Australian dollars.

### Statistical Analysis

Analyses were performed in RStudio with R (version 3.6.3; R Foundation for Statistical Computing). Descriptive analyses were conducted using Facebook and Instagram analytics data to assess how study advertisements performed and using Qualtrics survey data to determine rates of screened, eligible, and completed participants. A general linear model with pairwise post hoc estimates was used to compare the effectiveness of different methods in recruiting and retaining participants. For each recruitment pathway (Facebook and Instagram advertisement or post, other, and website of lead institution), Fischer exact tests were conducted to determine if gender, student status (domestic or international), and student level (undergraduate or postgraduate) of participants changed significantly as a result of dropout across different stages of the trial. To test for differences in age, Kruskal-Wallis tests were performed. For the purpose of comparing cost-effectiveness between COVID-19 lockdown and post–COVID-19 lockdown, pilot 1 and pilot 2 were classified in the former category and minitrials 1-6 fell into the latter. Inferential statistics were performed using Wilcoxon tests.

### Ethical Considerations

Ethical approval was sought and obtained from the University of New South Wales Sydney Human Research Ethics Committee (HC200466). The trial sponsor is the University of New South Wales Sydney. The trial is registered with the Australian New Zealand Clinical Trials Registry (Pilot trials: ACTRN12621001092886; Mini-trials 1-6: ACTRN12621001223820). Informed consent was obtained from each trial participant, and the collected data were stored in a deidentified format. Each participant received Aus $30 [US $18.65] in gift cards.

## Results

### Demographics

In total, 1314 participants were recruited for 2 pilot trials and 6 minitrials. Participants had a mean age of 22.79 (SD 4.71) years, predominately female (n=1079, 82.1%), domestic (n=1226, 93.3%), and undergraduate students (n=1045, 79.5%). Participants were recruited from 72 tertiary education institutions from across Australia (for the top 10 institutions; Table S1 in Multimedia Appendix 1).

### Breakdown by Recruitment Methods

For the 2 pilot trials and 6 minitrials, 3638 students attempted the screening survey. The majority of students who screened for the trials were recruited via social media advertising or posts via Facebook (n=1690, 47%) and Instagram (n=922, 25%). Of the 1314 students (36%) eligible to participate in the study, the vast majority were recruited via social media advertising or posts (n=1203, 92%). Of the 1058 students (81% of those eligible) who provided baseline data, similar proportions were recruited via social media advertising or posts. Of the 982 students (75% of those eligible) who provided midpoint data, recruitment via social media remained the most fruitful (n=889, 91%). Of the 862 students (66% of those eligible) who completed a trial, 90 percent of students were recruited via social media advertising or posts. In terms of participant retention, see Table 3 for a detailed breakdown by recruitment method.

**Table 3:** Breakdown of participants by recruitment pathway and trial retention.^a^

Recruitment pathway	Number of students retained in the trial				
	Attempted screening (N=3638), n	Screened eligible (n=1314), n (%)	Completed baseline (n=1058), n (%)	Completed midpoint (n=982), n (%)	Completed a trial (n=862), n (%)
Facebook advertisement or post	1690	810 (47.9)	644 (38.1)	597 (35.3)	518 (30.7)
Instagram advertisement or post	922	393 (42.6)	316 (34.3)	292 (31.7)	257 (27.9)
Other	107	61 (57)	55 (51.4)	52 (48.6)	49 (45.8)
Website of lead institution	72	50 (69.4)	43 (59.7)	41 (56.9)	38 (52.7)
Survey not completed	847	—^b^	—	—	—

^a^Other included class or event, email, health professional, poster or flyer, press or media. Retention percentages were calculated based on the number of participants who attempted screening.

^b^Not applicable.

Pairwise comparisons revealed that the lead institution’s website was more effective in recruiting eligible participants compared to Facebook (*z*=3.47; *P*=.003) and Instagram (*z*=4.23; *P*<.001). No statistically significant difference was found between website recruitment and other methods (eg, email, poster or flyer) in recruiting eligible participants. No statistically significant differences were found between recruitment methods in retaining participants at baseline, at mid-point, and at study completion (Tables S2-S9 in Multimedia Appendix 1). To assess possible sample selection effects by recruitment pathway, characteristics of participants who dropped out at each trial stage were examined. No statistically significant age, sex, and student level (undergraduate vs postgraduate) differences were found for recruitment by Facebook, Instagram, others, and the website of the lead institution (Tables S10-S13 in Multimedia Appendix 1). For recruitment by Facebook, statistically significant differences were found for student status (domestic vs international*; P*=.001; Table S10 in Multimedia Appendix 1). No such differences were found for Instagram, others, and the website of the lead institution (Tables S11-S13 in Multimedia Appendix 1).

### Facebook and Instagram Recruitment

Across the 2 pilot trials and 6 minitrials, Aus $7571.72 (US $4707.72) was spent on social media recruitment via Facebook and Instagram advertising, at an average of Aus $946.47 (US $588.47; SE Aus $160.99 [US $100.10]) per trial. A total of 1,119,661 impressions were counted, at an average of 139,958 (SE 27,927) impressions per trial. The performance of study advertisements was measured by the number of clicks generated that led potential participants from the social media platform to the Vibe Up recruitment page. See Figures S1 and S2 in Multimedia Appendix 2 for the top-performing Vibe Up advertisements. During the recruitment drives, 825,635 people were reached, at an average of 103,204 people (SE 17,569) per trial. 8138 clicks were registered on the study website (mean 1017, SE 71), with 7858 being unique link clicks (mean 982, SE 68). The overall click-through rate was 0.73. See Table 4 for the detailed breakdown of participant engagement by trial, during and post–COVID-19 lockdown.

**Table 4:** Breakdown of trial costs and engagement metrics.

Trial (stage)	Amount spent (Aus $^a^)	Impressions, n	Reach, n	Clicks, n	Unique clicks, n	Click-through rate
Pilot 1(lockdown)	445.98	82,266	65,654	1227	1155	1.49
Pilot 2 (lockdown)	254.11	39,709	31,592	699	669	1.76
Trial 1 (postlockdown)	850.00	91,322	71,994	1142	1112	1.25
Trial 2 (postlockdown)	999.70	237,201	150,766	1197	1156	0.50
Trial 3 (postlockdown)	1100.00	247,540	175,161	1203	1168	0.49
Trial 4 (postlockdown)	1750.80	207,799	146,423	967	937	0.47
Trial 5 (postlockdown)	1095.12	99,285	81,652	850	824	0.86
Trial 6 (postlockdown)	1076.01	114,539	102,393	853	837	0.74
Total	7571.72	1,119,661	825,635	8138	7858	0.73

^a^Aus $1=US $0.62.

### Cost-Effectiveness of Trial Recruitment

On average, it costs Aus $2.28 (US $1.42; SE Aus $0.49 [US $0.30]) per screened participant, Aus $6.59 (US $4.10; SE Aus $1.39 [US $0.86]) per eligible participant, Aus $7.78 (US $4.84; SE Aus $1.64 [US $1.02]) per participant who downloaded the study mobile app, and Aus $10.01 (US $6.22; SE Aus $2.20 [US $1.37]) per completion. See Table S16 in Multimedia Appendix 1 for the breakdown of costs by trial.

### Impacts of COVID-19 Lockdowns

Wilcoxon tests were carried out to compare costs incurred per link click between COVID-19 lockdown (pilot 1 and pilot 2), and post–COVID-19 lockdown (minitrials 1-6). Significant differences were found in the cost per click (lockdown: median Aus $0.35 [US $0.22], IQR Aus $0.27-$0.47 [US $0.17-$0.29]; postlockdown: median Aus $1.00 [US $0.62], IQR Aus $0.70-$1.47 [US $0.44-$0.92]; W=9087; *P*<.001; Table S14 in Multimedia Appendix 1). As recruitment drives occurred in short bursts (3-7 days as opposed to weeks or months for other recruitment campaigns), we compared lockdown and postlockdown costs using hourly spending on Facebook and Instagram advertising. Differences between lockdown and postlockdown on the amount spent per hour to reach the target sample size were also statistically significant (lockdown: median Aus $4.75 [US $2.95], IQR Aus $1.94-$6.34 [US $1.22-$3.97]; postlockdown: median Aus $13.29 [US $8.26], IQR Aus $4.70-$25.31 [US $2.95-$15.87]; W=16044; *P*<.001; Table S15 in Multimedia Appendix 1).

## Discussion

### Principal Findings

With the increasing adoption of remote clinical trials in digital mental health research, and the uptake of the AI-driven adaptive trial methodology, strategies to optimize timely recruitment of suitable participants warrant investigation. Advancements in the way online social media platforms can be manipulated to reach target audiences faster and cheaper provided further impetus for this study. The effectiveness of participant recruitment via Facebook and Instagram was compared with traditional methods such as mailing lists and study flyers or posters. Results suggest that Facebook and Instagram are effective in recruiting young adults for remote digital mental health trials. Social media advertising outperformed more traditional methods in the total number of eligible participants recruited. However, we also found that compared to Facebook and Instagram, recruitment via the lead institution’s website was more effective in yielding eligible participants from those who completed screening. It may be the case that participants recruited while browsing the institution’s website (a mental health research institute) were actively seeking information to manage mental health concerns, and thus more likely to be eligible for the study than someone shown the study advertisement via social media because they fit the target age group. In addition, although not statistically significant, the completion rate was higher for participants recruited via the website. This could be explained by the familiarity, trust, and support of the mental health research carried out by the lead institution and suggests that this recruitment pathway can be effective for trial enrollment and importantly, trial completion. Given that the vast majority of eligible participants were recruited via social media advertising (92%) compared to website recruitment (4%), if funds are available, it would still make sense to include social media in the recruitment strategy to recruit young people for digital mental health trials. In terms of participant retention from study baseline to completion, there was no stand-out recruitment method. Similar to the number of eligible participants, the sheer volume of completers recruited via social media (90%) compared to traditional methods, provides a rationale for including this method in participant recruitment strategies. We did not find evidence of a sample selection effect due to the recruitment methods used. However, similar trials in the future should identify strategies to attract more international students. For example, targeted social media advertising for this student group and liaising with student leaders to disseminate trial information to club or society members.

In terms of cost-effectiveness, this study provided further evidence to support the benefits of using social media to recruit young adults for mental health research participation. Our results (Aus $7.78 [US $4.84] per participant app download and Aus $10.01 [US $6.22] per trial completion) compared favorably with the Mood Memos Study (Aus $19.89 [US $12.37] per recruited participant) [6], a health survey study targeting young females (US $20 per compliant participant) [12], a health intervention study of 334 young Australian adults (Aus $105.77 [US $65.76] per recruited participant) [25], diabetes self-management clinical trial for young adults (US $334 per enrolled participant) [14], and a review that calculated estimated median recruitment costs for RCTs in mental health research (US $42.82) [10]. Even though costs per completion for our trial were higher than studies conducted by Lee et al [8] (Aus $2.81 [US $1.75] per completion), Ash et al [26] (Aus $6.07 [US $3.77] per completion) [26], and Batterham [9] (Aus $1.51 [US $0.94] per completion), additional costs are explained by the nature of research activities involved; one-off survey completions, as opposed to completing a 29-day trial involving brief mental health interventions in Vibe Up. In comparison to an RCT that recruited 560 Danes aged 15-25 years to evaluate an online youth mental health promotion service [13], US $4.20 per baseline survey completion reported for this trial was almost identical to costs per eligible participant for Vibe Up (Aus $6.59 [US $4.10]).

More than 1.1 million impressions were counted during our recruitment drives, reaching 825,635 people. These are numbers difficult to achieve in a short period of time using flyers or posters, class announcements, and mailing lists. The ability to track the number of clicks on the study website (n=8138), and determine if clicks were by different individuals (7858 unique clicks), in addition to the ability to alter targeting setup (eg, tailoring advertisements to male students), makes social media recruitment via Facebook and Instagram an attractive proposition for researchers looking to recruit a large sample within a limited period of time. Further, click-through rates provided valuable insights into how engaging the study advertisements are to the target audience, allowing fine-tuning of recruitment materials during recruitment drives to maximize participant sign-up. According to WordStream [27], a good click-through rate for Facebook advertising averages approximately 0.90%. This study achieved an overall click-through rate of 0.73%, with the highest rates achieved during the COVID-19 lockdown (pilot 1: 1.49 and pilot 2: 1.76). It is important to acknowledge that the success in recruiting young people for our trial also leveraged the well-established and trusted brand of the lead institution in the mental health space in Australia.

A spike in costs for minitrial 4 warranted our attention. Up until then, a “link click” objective was used wherein Facebook and Instagram directed the study advertisements toward individuals most likely to click on them. A change to the “landing page view” objective for trial 4, where individuals more likely to load the page content were targeted, resulted in higher costs. It was a tactic that did not pay off as Facebook and Instagram needed time and more money to relearn the change in objective. For subsequent trials, a conversion pixel on the “GET STARTED” button was implemented, targeting people most likely to participate. This was a cost-saving strategy, homing in on people more likely to take action. Although the ability to change strategies demonstrated advancements in social media recruitment to reach specific target samples, it does come with additional financial costs that researchers should be aware of. In addition, fraudulent participants have increasingly infiltrated research recruitment via social media, especially trials offering monetary reimbursement. The current study implemented several safeguards including the use of time-based one-time password for app downloads and manual checking of participant details before inclusion in the study.

The first 2 recruitment drives coinciding with the COVID-19 lockdowns provided us with the unique opportunity to assess whether social media recruitment was more cost-effective during the lockdown period compared to postlockdown. Comparisons between the 2 periods found that fewer funds were needed during lockdown to trigger interest from prospective participants and result in sign-ups. The COVID lockdown may have facilitated recruitment, with students taking the opportunity to learn new, evidence-based coping strategies for psychological distress offered by the trial. The planned recruitment period of 2 weeks was reduced to 7 days, with almost 300 participants recruited for the first pilot trial. Adjustments were made for pilot 2 (7-day recruitment period); however, intake was closed after 3 days with sign-ups exceeding the target of 120-150 participants. COVID-19 lockdown was the most cost-effective recruitment period, with fewer funds required per screened participant, per eligible participant, per app download, and per completed participant. The psychological impact of the lockdown may have prompted students to seek ways to improve their mental well-being. Even though recruitment periods for subsequent trials (postlockdown) remained consistent (3-5 days), more funds were expended to reach the target sample size. Costs per completed participant increased to between Aus $8 (US $4.97) and Aus $21 (US $13.06; compared to under Aus $3 [US $1.87] for the pilot trials). These opportunistic findings suggest that recruitment of young people may yield better results during their “downtime,” for example, during semester breaks or festive seasons, when they have the capacity to contribute to research. In addition, recruitment materials need to include information on benefits to participants (for example, providing an opportunity to learn evidence-based coping strategies), and screening needs to exclude those unable to commit for the entire study (for example, asking participants to indicate major disruptions or events in the next 2 months which may make it difficult to take part in the study) to help boost retention rates.

### Limitations

First, we were unable to accurately assign a monetary value to the traditional recruitment methods used for our trials (eg, poster or flyer and classroom announcements) so no direct comparisons were made on cost-effectiveness with social media recruitment. As a result, we are unable to comment on whether one recruitment method is more cost-effective than another in this study. Instead, the costs of social media recruitment were compared with previously published data to help determine if this method should be pursued in future mental health AI-driven adaptive trials. Secondly, we struggled to correct the sex imbalance within our sample. This is consistent with the recruitment literature [28,29]. Even though more funds were allocated to the recruitment of male participants from main trial 4 onwards, we were unable to correct the skew. For future studies, user testing of tailored advertisements for male participants should be considered before the start of recruitment campaigns. Next, given that our participants were recruited from all over Australia, it was difficult to isolate which participants were in lockdown, as lockdown periods varied across the country. We have categorized our trials based on dates published online by the Australian government [30]. Finally, the young people referred to in our study are all tertiary education students. Their characteristics and inclination to participate in research may differ from others in this group (eg, in full-time employment). Hence, our findings may not be generalizable to all young people in Australia but are likely to reflect those currently attending an Australian tertiary education institution.

### Conclusions

Social media advertising on Facebook and Instagram is a productive method to recruit young people for AI-driven adaptive digital mental health intervention trials. Given that repeated recruitment drives are necessary for this trial methodology (in our case, once every month for 2 pilot trials and 6 minitrials), we found social media to be more effective in reaching potential participants all over Australia than traditional recruitment methods such as mailing lists and flyers or posters. In addition, advertising costs from this study compared favorably with other large-scale traditional trials, providing evidence that social media advertising can be effective for participant recruitment in future AI-driven adaptive trials. Perhaps a reflection of the added distress experienced by young people, online advertising costs were low during COVID-19 lockdowns and increased post lockdown. Based on our experience, we encourage other researchers interested in running AI-driven adaptive trials to consider the use of social media advertising to recruit participants.

## Appendix

Multimedia Appendix 1 Additional tables.

Multimedia Appendix 2 Vibe Up study advertisements.

## Abbreviations

AI: artificial intelligence
RCT: randomized controlled trial
