# Supplementary material for: Recruiting Young People for Digital Mental Health Research: Lessons From an AI-Driven Adaptive Trial
Source: J Med Internet Res. 2025 Jan 14;27:e60413. doi: 10.2196/60413 (PMC11775482; doi:10.2196/60413)
Supplement: Multimedia Appendix 1 [file jmir_v27i1e60413_app1.docx]

**Table S1.** Origin of participants: Top 10 Australian tertiary education institutions

| Institution | Number of participants recruited |
| --- | --- |
| The University of Melbourne | 67 |
| University of New South Wales | 65 |
| Monash University | 59 |
| The University of Queensland | 56 |
| Deakin University | 55 |
| The University of Sydney | 55 |
| Australian National University | 52 |
| Griffith University | 49 |
| Macquarie University | 41 |
| University of Newcastle | 41 |

| **Table S2.** Eligible participants by recruitment pathways | | | | |
| --- | --- | --- | --- | --- |
| Recruitment pathway | M | SE | LL | UL |
| Lead Institution Website | 0.70 | 0.05 | 0.58 | 0.79 |
| Facebook | 0.48 | 0.01 | 0.46 | 0.50 |
| Instagram | 0.43 | 0.02 | 0.40 | 0.46 |
| Other | 0.57 | 0.05 | 0.48 | 0.66 |
| *Note.* 95% CI. | | | | |

| **Table S3.** Post-hoc comparisons of recruitment pathways (Eligible participants) | | | | |
| --- | --- | --- | --- | --- |
| Contrast | Estimate | SE | *z* | *P* |
| Website / Facebook | 2.47 | 0.64 | 3.47 | .003 |
| Website / Instagram | 3.06 | 0.81 | 4.23 | <.001 |
| Website / Other | 1.71 | 0.55 | 1.67 | .338 |
| Facebook / Instagram | 1.24 | 0.10 | 2.60 | .046 |
| Facebook / Other | 0.69 | 0.14 | -1.81 | .267 |
| Instagram / Other | 0.56 | 0.12 | -2.81 | .026 |
| *Note.* df = 1. *P* value adjusted using Tukey’s method. Tests performed on the log odds ratio scale. | | | | |

| **Table S4.** Baseline completion by recruitment pathways | | | | |
| --- | --- | --- | --- | --- |
| Recruitment pathway | M | SE | LL | UL |
| Lead Institution Website | 0.86 | 0.05 | 0.73 | 0.93 |
| Facebook | 0.80 | 0.01 | 0.77 | 0.82 |
| Instagram | 0.80 | 0.02 | 0.76 | 0.84 |
| Other | 0.90 | 0.04 | 0.80 | 0.96 |
| *Note.* 95% CI. | | | | |

| **Table S5.** Post-hoc comparisons of recruitment pathways (Baseline completion) | | | | |
| --- | --- | --- | --- | --- |
| Contrast | Estimate | SE | *z* | *P* |
| BDI / Facebook | 1.58 | 0.66 | 1.10 | .69 |
| BDI / Instagram | 1.50 | 0.64 | 0.95 | .78 |
| BDI / Other | 0.67 | 0.40 | -0.68 | .91 |
| Facebook / Instagram | 0.95 | 0.15 | -0.37 | .98 |
| Facebook / Other | 0.42 | 0.19 | -1.96 | .20 |
| Instagram / Other | 0.45 | 0.20 | -1.79 | .28 |
| *Note.* df = 1. *P* value adjusted using Tukey’s method. Tests performed on the log odds ratio scale. | | | | |

| **Table S6.** Mid-point completion by recruitment pathways | | | | |
| --- | --- | --- | --- | --- |
| Recruitment pathway | M | SE | LL | UL |
| BDI | 0.96 | 0.03 | 0.84 | 0.99 |
| Facebook | 0.93 | 0.01 | 0.91 | 0.95 |
| Instagram | 0.93 | 0.01 | 0.90 | 0.95 |
| Other | 0.95 | 0.03 | 0.85 | 0.98 |
| *Note.* 95% CI. | | | | |

| **Table S7.** Post-hoc comparisons of recruitment pathways (Mid-point completion) | | | | |
| --- | --- | --- | --- | --- |
| Contrast | Estimate | SE | *z* | *P* |
| BDI / Facebook | 1.57 | 1.16 | 0.61 | .93 |
| BDI / Instagram | 1.63 | 1.23 | 0.65 | .92 |
| BDI / Other | 1.17 | 1.10 | 0.17 | .99 |
| Facebook / Instagram | 1.04 | 0.27 | 0.15 | .99 |
| Facebook / Other | 0.75 | 0.46 | -0.48 | .96 |
| Instagram / Other | 0.72 | 0.45 | -0.53 | .95 |
| *Note.* df = 1. *P* value adjusted using Tukey’s method. Tests performed on the log odds ratio scale. | | | | |

| **Table S8.** Trial completion by recruitment pathways | | | | |
| --- | --- | --- | --- | --- |
| Recruitment pathway | M | SE | LL | UL |
| BDI | 0.94 | 0.04 | 0.82 | 0.98 |
| Facebook | 0.89 | 0.01 | 0.87 | 0.91 |
| Instagram | 0.90 | 0.02 | 0.86 | 0.93 |
| Other | 0.95 | 0.03 | 0.85 | 0.98 |
| *Note.* 95% CI. | | | | |

| **Table S9.** Post-hoc comparisons of recruitment pathways (Trial completion) | | | | |
| --- | --- | --- | --- | --- |
| Contrast | Estimate | SE | *z* | *P* |
| BDI / Facebook | 1.76 | 1.07 | 0.93 | .79 |
| BDI / Instagram | 1.59 | 0.99 | 0.74 | .88 |
| BDI / Other | 0.78 | 0.66 | -0.29 | .99 |
| Facebook / Instagram | 0.90 | 0.19 | -0.48 | .96 |
| Facebook / Other | 0.45 | 0.27 | -1.34 | .54 |
| Instagram / Other | 0.49 | 0.31 | -1.14 | .66 |
| *Note.* df = 1. *P* value adjusted using Tukey’s method. Tests performed on the log odds ratio scale. | | | | |

**Table S10**. Comparing characteristics of participants who dropped out (Facebook)

| Variables | Levels | Eligible participants (N=810) | Eligible participants who failed to complete baseline (N=166) | Participants who completed baseline but failed to complete mid-point (N=47) | Participants who completed mid-point but failed to complete post (N=79) |
| --- | --- | --- | --- | --- | --- |
| Mean age (years) ^a^ |  | 23.01 (SD=4.25) | 22.95 (SD=4.46) | 22.72 (SD=2.84) | 22.17 (SD=3.47) |
| Gender ^b^ | Female | 686 (84.7%) | 138 (82.6%) | 34 (72%) | 61 (78%) |
|  | Male | 124 | 28 | 13 | 18 |
| Student status ^c^ | Domestic | 763 (94.2%) | 149 (89.2%) | 46 (98%) | 79 (100%) |
|  | International | 47 | 17 | 1 | 0 |
| Student level ^d^ | Undergraduate | 632 (78.0%) | 134 (80.2%) | 42 (89%) | 62 (79%) |
|  | Postgraduate | 178 | 32 | 5 | 17 |

^a^Kruskal-Wallis test, χ^2^ (2) =2.26, *P*=.32

^b^Fisher’s Exact test, *P*=.27

^c^Fisher’s Exact test, *P<.001*

^d^Fisher’s Exact test, *P*=.33

**Table S11**. Comparing characteristics of participants who dropped out (Instagram)

| Variables | Levels | Eligible participants (N=393) | Eligible participants who failed to complete baseline (N=77) | Participants who completed baseline but failed to complete mid-point (N=24) | Participants who completed mid-point but failed to complete post (N=35) |
| --- | --- | --- | --- | --- | --- |
| Mean age (years) ^a^ |  | 22.01 (SD=4.17) | 22.44 (SD=4.14) | 21.42 (SD=3.39) | 22.00 (SD=3.40) |
| Gender ^b^ | Female | 303 (76.9%) | 61 (78%) | 17 (71%) | 25 (71%) |
|  | Male | 90 | 16 | 7 | 10 |
| Student status ^c^ | Domestic | 365 (92.6%) | 70 (90%) | 23 (96%) | 33 (94%) |
|  | International | 28 | 7 | 1 | 2 |
| Student level ^d^ | Undergraduate | 323 (82.0%) | 65 (83%) | 19 (79%) | 33 (94%) |
|  | Postgraduate | 70 | 12 | 5 | 2 |

^a^Kruskal-Wallis test, χ^2^ (2) =1.16, *P*=.56

^b^Fisher’s Exact test, *P*=.63

^c^Fisher’s Exact test, *P*=.75

^d^Fisher’s Exact test, *P*=.17

**Table S12**. Comparing characteristics of participants who dropped out (Other ^a^)

| Variables | Levels | Eligible participants (N=61) | Eligible participants who failed to complete baseline (N=6) | Participants who completed baseline but failed to complete mid-point (N=3) | Participants who completed mid-point but failed to complete post (N=3) |
| --- | --- | --- | --- | --- | --- |
| Mean age (years) ^b^ |  | 21.95 (SD=3.69) | 21.50 (SD=1.97) | 18.33 (SD=0.58) | 22.67 (SD=4.62) |
| Gender ^c^ | Female | 47 (77%) | 3 (50%) | 100 (3:0) | 100 (3:0) |
|  | Male | 14 | 3 | 0 | 0 |
| Student status ^d^ | Domestic | 52 (85%) | 4 (67%) | 100 (3:0) | 100 (3:0) |
|  | International | 9 | 2 | 0 | 0 |
| Student level ^e^ | Undergraduate | 52 (85%) | 6 (100%) | 100 (3:0) | 100 (3:0) |
|  | Postgraduate | 9 | 0 | 0 | 0 |

*Note.* Interpret comparison tests with caution due to low count values

^a^Other included class or event, email, health professional, poster/flyer, press/media.

^b^Kruskal-Wallis test, χ^2^ (2) =5.95, *P*=.05

^c^Fisher’s Exact test, *P*=.34

^d^Fisher’s Exact test, *P*=.45

^e^Fisher’s Exact test, *P*=1

**Table S13**. Comparing characteristics of participants who dropped out (Website of lead institution)

| Variables | Levels | Eligible participants (N=50) | Eligible participants who failed to complete baseline (N=7) | Participants who completed baseline but failed to complete mid-point (N=2) | Participants who completed mid-point but failed to complete post (N=3) |
| --- | --- | --- | --- | --- | --- |
| Mean age (years) ^a^ |  | 25.54 (SD=8.20) | 21.86 (SD=2.04) | 24.00 (SD=5.66) | 22.67 (SD=3.21) |
| Gender ^b^ | Female | 88 (44:6) | 7 (100%) | 1 (50%) | 3 (100%) |
|  | Male | 6 | 0 | 1 | 0 |
| Student status ^c^ | Domestic | 94 (47:3) | 7 (100%) | 2 (100%) | 2 (67%) |
|  | International | 3 | 0 | 0 | 1 |
| Student level ^d^ | Undergraduate | 78 (39:11) | 5 (71%) | 1 (50%) | 3 (100%) |
|  | Postgraduate | 11 | 2 | 1 | 0 |

*Note.* Interpret comparison tests with caution due to low count values

^a^Kruskal-Wallis test, χ^2^ (2) =0.48, *P*=.79

^b^Fisher’s Exact test, *P*=.17

^c^Fisher’s Exact test, *P*=.42

^d^Fisher’s Exact test, *P*=.73

**Table S14**. Costs incurred per click (Lockdown vs. Post-lockdown)

| Stage | Corresponding trial | Mean cost per click | Median cost per click | SD cost per click | SEM | IQR |
| --- | --- | --- | --- | --- | --- | --- |
| Lockdown | Pilot 1 + Pilot 2 | 0.40 | 0.35 | 0.24 | .02 | 0.20 |
| Post-lockdown | Trial 1 – 6 | 1.17 | 1.00 | 0.92 | .04 | 0.77 |

*Note.* Lockdown for major cities in Australia. Wilcoxon test (W=9087, *P*<0.001)

**Table S15.** Costs incurred per hour to reach target sample size (Lockdown vs. Post-lockdown)

| Stage | Corresponding trial | Mean cost | Median cost | SD cost | SEM | IQR |
| --- | --- | --- | --- | --- | --- | --- |
| Lockdown | Pilot 1 + Pilot 2 | 4.46 | 4.75 | 2.46 | .20 | 4.4 |
| Post-lockdown | Trial 1 - 6 | 15.83 | 13.29 | 12.63 | .61 | 20.61 |

*Note.* Lockdown for major cities in Australia. Wilcoxon test (W=16044, *P*<0.001)

**Table S16.** Cost Effectiveness of trial recruitment using social media

| Trial | Recruitment dates | Recruitment duration (days) | States in lockdown | Eligible participants recruited per day | Number of screened participants/Cost per screened participant (AUD$) | Number of eligible participants/Cost per eligible participant (AUD$) | Number of participants who downloaded app/Cost per download (AUD$) | Number of completed participants/ Cost per completion (AUD$) |
| --- | --- | --- | --- | --- | --- | --- | --- | --- |
| Pilot 1 | 28^th^ June – 4^th^ July 2021 | 7 | NSW, NT, QLD, VIC, WA | 40 | 688/0.65 | 282/1.58 | 216/2.06 | 165/2.70 |
| Pilot 2 | 30^th^ August – 1^st^ September 2021 | 3 | ACT, NSW, VIC | 60 | 426/0.60 | 180/1.41 | 150/1.69 | 120/2.12 |
| Trial 1 | 29^th^ October – 2^nd^ November 2021 | 5 | No lockdown | 26 | 373/2.28 | 130/6.54 | 116/7.33 | 87/9.77 |
| Trial 2 | 10^th^ January – 13^th^ January 2022 | 4 | No lockdown | 30 | 374/2.67 | 118/8.47 | 94/10.64 | 70/14.28 |
| Trial 3 | 14^th^ February – 16^th^ February 2022 | 3 | No lockdown | 43 | 394/2.79 | 128/8.59 | 108/10.19 | 89/12.36 |
| Trial 4 | 7^th^ March – 10^th^ March 2022 | 4 | No lockdown | 32 | 347/5.05 | 129/13.57 | 110/15.92 | 82/21.35 |
| Trial 5 | 26^th^ April – 28^th^ April 2022 | 3 | No lockdown | 59 | 525/2.09 | 178/6.15 | 157/6.98 | 134/8.17 |
| Trial 6 | 16^th^ May – 18^th^ May 2022 | 3 | No lockdown | 56 | 511/2.11 | 169/6.37 | 145/7.42 | 115/9.36 |
