# Supplementary figures and images for: Recruiting Young People for Digital Mental Health Research: Lessons From an AI-Driven Adaptive Trial
Source: J Med Internet Res. 2025 Jan 14;27:e60413. doi: 10.2196/60413 (PMC11775482; doi:10.2196/60413)

**Figure S1.** Top performing Vibe Up advertisement 1


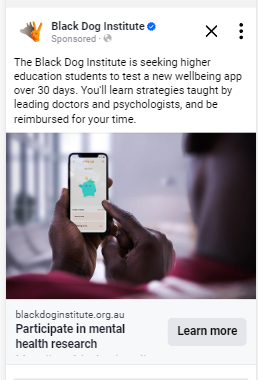


**Figure S2**. Top performing Vibe Up advertisement 2


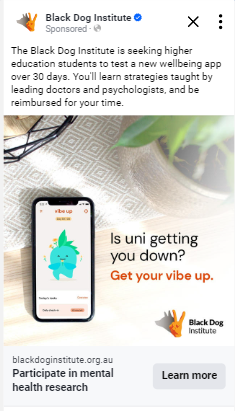

Supplement: Multimedia Appendix 2 [file jmir_v27i1e60413_app2.docx]
